# Pheochromocytoma and paraganglioma: implications of germline mutation investigation for treatment, screening, and surveillance

**DOI:** 10.20945/2359-3997000000145

**Published:** 2019-07-11

**Authors:** Ana Milena Gómez, Diogo Cordeiro Soares, Alexandre André Balieiro Costa, Daniele Paixão Pereira, Maria Isabel Achatz, Maria Nirvana Formiga

**Affiliations:** 1 Hospital Universitario San Ignacio Bogotá Colombia Hospital Universitario San Ignacio, Bogotá, Colombia; 2 Departamento de Oncogenética A.C. Camargo Cancer Center São Paulo SP Brasil Departamento de Oncogenética, A.C. Camargo Cancer Center, São Paulo, SP, Brasil; 3 Hospital Sírio-Libanês Centro de Oncologia Hospital Sírio-Libanês São Paulo SP Brasil Centro de Oncologia, Hospital Sírio-Libanês, São Paulo, SP, Brasil

**Keywords:** Pheochromocytoma, paraganglioma, germline mutation, succinate dehydrogenase complex, screening

## Abstract

**Objective:**

Paraganglioma (PGL) and pheochromocytoma (PCC) are rare neuroendocrine tumors that were considered to be predominantly sporadic. However, with the identification of novel susceptibility genes over the last decade, it is currently estimated that up to 40% of cases can occur in the context of a hereditary syndrome. We aimed to characterize PGL/PCC families to exemplify the different scenarios in which hereditary syndromes can be suspected and to emphasize the importance for patients and their families of making an opportune genetic diagnosis.

**Materials and methods:**

Retrospective analysis of patients diagnosed with PGL/PCC. Germline mutations were studied using next-generation sequencing panels including SDHA, SDHB, SDHC and SDHD. Clinical data were collected from clinical records, and all patients received genetic counseling.

**Results:**

We describe 4 families with PGL/PCC and germline mutations in SDH complex genes. 2 families have SDHB mutations and 2 SDHD mutations. The clinical presentation of the patients and their families was heterogeneous, with some being atypical according to the literature.

**Conclusions:**

PGL/PCC are more commonly associated with a germline mutation than any other cancer type, therefore, all individuals with these types of tumors should undergo genetic risk evaluation. NGS multigene panel testing is a cost-effective approach given the overlapping phenotypes. Individuals with germline mutations associated with PGL/PCC should undergo lifelong clinical, biochemical and imaging surveillance and their families should undergo genetic counseling. For all these reasons, it is critical that all medical staff can suspect and diagnose these inherited cancer predisposition syndromes.

## INTRODUCTION

Paraganglioma (PGL) and pheochromocytoma (PCC) are rare neuroendocrine tumors of the autonomic nervous system that occur in the extra-adrenal ganglia and adrenal medulla, respectively. The combined annual incidence of PGL/PCC has been estimated to be 1/300,000 with the peak age of occurrence being in the third to fifth decade of life without a gender difference ( 1 ).

PCCs, which can be described as a form of sympathetic PGL and may be discovered incidentally as a mass on magnetic resonance imaging (MRI) or computed tomography. PCC symptoms are generally attributable to catecholamine hypersecretion (i.e., hypertension, headache, palpitations, excessive sweating, and anxiety) or mass effects ( 2 ). Meanwhile, PGLs of the skull base and neck are generally associated with parasympathetic nervous system structures and without effects on catecholamine secretion. Thus their symptoms are produced by mass effects.

Previously, PGL/PCC tumors were considered to be predominantly sporadic. However, the recently expanded availability of next generation sequencing methodology has led to the identification of novel PGL/PCC susceptibility genes. It appears that some 40% of cases are linked to an autosomal-dominant hereditary syndrome ( 3 - 6 ). Even among cases considered to be sporadic with no family history, studies have shown that 11–24% have a germline mutation affecting one of 16 implicated PGL/PCC susceptibility genes ( 7 - 10 ). PGL/PCC development has been related to mutations in three genes that cause well-known cancer susceptibility syndromes: *VHL* (von hippel-lindau tumor supressor), *NF1* (neurofibromin 1), and *RET* (ret proto-oncogene). The syndromes associated with mutations in these three genes are Von Hippel-Lindau disease (VHL), neurofibromatosis type 1 (NF1), and Multiple Endocrine Neoplasia type 2 (MEN2), respectively.

Additionally, mutations affecting succinate dehydrogenase (SDH) complex subunit genes ( *SDHA, SDHB, SDHC, SDHD* ) and one of the SDH complex factor genes ( *SDHAF2* ) are associated with a predisposition to PGL/PCC with variable risks ( 11 ). Of these genes, *SDHB* is the most commonly mutated, resulting mainly in extra-adrenal sympathetic tumors with a high risk of metastasis ( 12 , 13 ), followed by *SDHD* , which has been associated with parasympathetic head and neck PGL. Interestingly, only paternally inherited *SDHD* mutations cause a disease phenotype, with rare exceptions ( 14 - 16 ). There are others tumor risks associated with SDH complex gene mutations, including gastrointestinal stromal tumors (GISTs) in Carney-Stratakis syndrome (dyad of PGL/PCC and GIST related to *SDHB* , *SDHC* , and *SDHD* mutations) and renal cell carcinoma, which has been described in some families with *SDHB* mutations ( 17 , 18 ). Although the Carney's triad (PGL/PCC and GIST, together with pulmonary chondroma) has been considered to be a sporadic condition, Carney's triad patients with germline mutations in *SDHA* , *SDHB* , and *SDHC* have been described recently ( 19 ). Germline mutations in *SDH* have also been found in patients with pituitary tumors, non-medullary thyroid cancer, neuroblastoma, adrenal hyperplasia, and testicular seminoma ( 20 ), though the significance of these findings is unclear.

Other putative PGL/PCC susceptibility genes described in the literature include *MAX* and *TMEM127* , both of which were found in association with PCC ( 21 , 22 ). Mutations in *FH* , *EGLN1* , *EGLN2* , *MDH2* , *EPAS1* , and *KIF1B* have also been found in small numbers of cases with a possibly modest pathogenic contribution ( 6 ). Mutations in *SDHAF2* and *MAX* genes exhibit parent-of-origin effects similar to *SDHD* mutations, as described above.

Given the high rate of inherited mutations associated with PGL/PCC, hereditary syndromes should be considered in all individuals with PGL and/or PCC, prioritizing those with the following findings: early onset (< 45 years old), recurrent or malignant tumors, multiple PGL/PCC or bilateral adrenal tumors and family history ( 23 ). The Endocrine Society recommends considering genetic testing in all patients with PGL/PCC with a clinical feature-driven algorithm based on tumor location and catecholamine biochemical phenotype to guide the selection of genes to the be tested. ( 23 ) Nevertheless, as panel gene testing is becoming more cost effective, it is replacing single-gene testing in most centers.

Based on the above, and considering the importance of this topic, we present four families with PGL/PCC and mutations in SDH complex subunit genes. Our aim was to exemplify different scenarios in which hereditary syndromes can be suspected. We emphasize the importance of making an opportune genetic diagnosis for patients and their families.

## MATERIALS AND METHODS

We conducted a retrospective analysis, collecting clinical phenotype and genetic data of patients diagnosed with PGL/PCC. The patients were followed-up in the Department of Oncogenetics at A. C. Camargo Cancer Center, whose Ethics Committee approved this study.

DNA extracted from peripheral blood samples obtained from all probands were submitted to mutation analyses in a next-generation sequencing platform with a physician-ordered basic panel including *SDHA, SDHB, SDHC* , and *SDHD* at a commercial Genetics Laboratory in Brazil. Variants found in target genes were classified based on guidelines of American College of Medical Genetics and Genomics, including their five-category system: class 1 (clearly not pathogenic), class 2 (unlikely to be pathogenic), class 3 (unknown significance), class 4 (likely to be pathogenic), and class 5 (clearly pathogenic) ( 24 , 25 ).

## RESULTS

**Figure 1:**
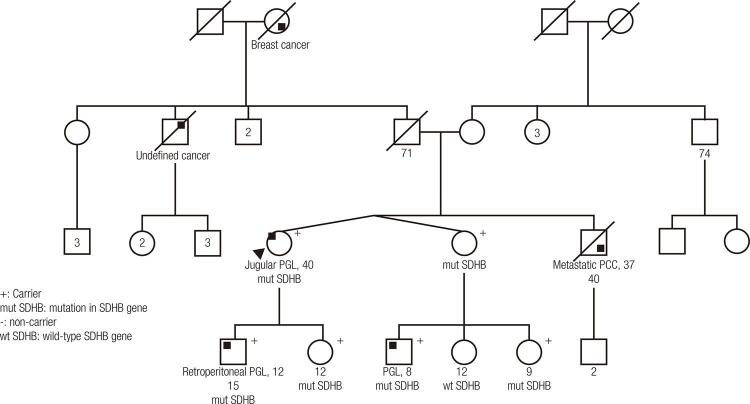
Pedigree of family with mutation in *SDHB gene*

In family 1, the index case patient had a jugular PGL that appeared at 40 years of age. Her brother had been diagnosed with metastatic PCC at 37 years old and died at 40 years old. The index case patient underwent genetic counseling and testing, which revealed a heterozygous c.393delA mutation (p.His132Thrfs*4) in *SDHB* that resulted in an adenine deletion that shifted the reading frame and created a premature stop codon. Her asymptomatic son and daughter were both found to be carriers. Imaging studies revealed a retroperitoneal PGL in the son, who was 12 years old. The proband’s sister was positive for the mutation but was unaffected at 43 years of age. That sister’s oldest son was positive for the mutation and diagnosed with PGL at 8 years old. The proband’s sister’s youngest daughter was also confirmed to carry the mutation, but was asymptomatic at the age of 9 years ( Figure 1 ).

In family 2, the index case patient was diagnosed with a carotid body PGL at 33 years of age. She underwent genetic counseling and testing that revealed a heterozygous c.689G>A point mutation (p.Arg230His) in *SDHB* . This mutation has been reported previously to be pathogenic and has been observed in individuals and families with early-onset, multifocal, and/or malignant PGL/PCC ( 26 ). The proband’s asymptomatic brother tested negative for the mutation.

**Figure 2:**
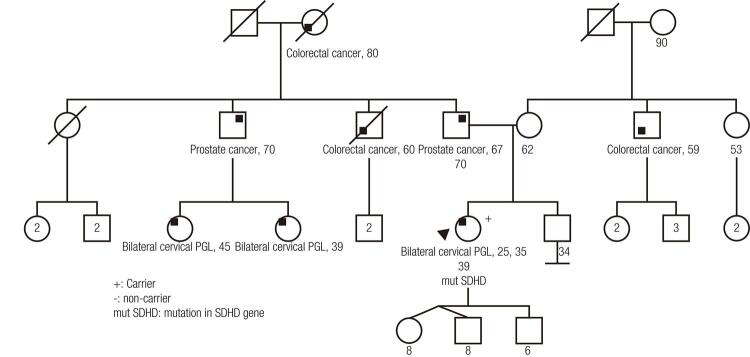
Pedigree of family with mutation in *SDHD gene*

In family 3, the index case patient had bilateral carotid body PGLs at 25 years of age. At the time of that diagnosis, she underwent excision surgery. Subsequently, she experienced PGL relapse bilaterally at 35 years old. She underwent genetic counseling and testing that revealed a heterozygous c.3G>C point mutation (p.Met1Ile) in *SDHD* , which had been described as pathogenic ( 27 ). Two paternal cousins had bilateral cervical PGLs at the ages of 39 years and 45 years; neither has been evaluated for the mutation. The asymptomatic children of the proband have not been tested yet either ( Figure 2 ).

In family 4, the index case patient had a PCC at 38 years old, and his son was diagnosed with metastatic PCC affecting a lung at the age of 11 years and bilateral carotid body PGLs at the age of 13 years. Th proband underwent genetic counseling and testing that revealed a heterozygous c.361C>T point mutation (p.Gln121*) in *SDHD* that resulted in a premature translational stop signal in the last exon of *SDHD* mRNA. The identified mutation had been classified previously as likely pathogenic (class 4 ), and was reclassified in last year as pathogenic (Class 5). The proband’s sister had jugular PGL at 38 years of age, but neither she nor the proband’s aforementioned son have been tested for the mutation. The proband indicated that his mother, a maternal uncle, and a maternal cousin had been diagnosed with PGL, but no history records were available to confirm this information ( Table 1 ).

**Table 1:** Comparison of families with germline mutations in SDH complex genes

Family ID	Mutated gene	Mutation (DNA)	Mutation (protein)	Earliest age of onset (yr)	Ages of all patients at diagnosis (yr)	Types of tumors in family	Unexpected Clinical Features
F1	SDHB	c.393delA	p.His132ThrfsX4	8	8, 12, 37, 40	Abdominal and HN PGL PCC (M)	Index case with HN PGL*
F2	SDHB	c.689G>A	p.Arg230His	33	33	HN PGL	Index case with HN PGL**
F3	SDHD	c.3G>C	p.Met1Ile	25	25, 39, 45	HN PGL	Typically presentation and paternally inherited transmission
F4	SDHD	c.361C>T	p.Gln121*	11	11, 13, 38, 38	PCC(M), HN PGL	Unusual malignant PCC and maternally inherited transmission

* Unusual for *SDHB* gene mutation. ** Unique presentation in the family, more frequent in sporadic scenario.

ID: identification; PCC: pheochromocytoma; PGL: paraganglioma; (M): metastases; HN: head and neck.

Most of the PGL/PCC cases identified in the four study families (11/12, 92%) were diagnosed in people younger than 40 years old. Recurrent, bilateral, multiple, and malignant tumors were prevalent in 8.3%, 33.3%, 16.7%, and 16.7% of the cases in families 1–4, respectively. No probands or family members were diagnosed with GIST or renal cell carcinoma. Regarding catecholamine biochemical parameters, none of our patients had abnormal biochemical findings at initial investigations or follow ups.

## DISCUSSION

Although rare, PGL/PCC show the highest degree of heritability among neoplasias ( 28 ). Genetic testing should be considered in all patients with these tumors because at least 30~40% of all patients with PGL/PCC have pathogenic germline mutations, and up to 50% of metastatic PGL/PCC may be associated with *SDHB* mutations. The confirmation of a hereditary syndrome in a patient makes it possible to diagnose and treat affected relatives early, reducing morbidity and mortality related to the disease ( 5 , 23 , 29 ). If clinical diagnosis of an established cancer susceptibility syndrome (e.g. VHL, NF1, or MEN2) can be made based on the presence of other cardinal manifestations, direct gene testing is recommended for molecular confirmation ( 11 ).

In non-syndromic forms, the patient’s biochemical profile and clinical phenotype can guide gene testing prioritization based on established genotype-phenotype correlations ( 30 , 31 ). Overlapping of clinical phenotypes may complicate prioritization in gene-to-gene approaches. However, next-generation sequencing is being integrated with rapidity into clinical molecular diagnostics, including those for hereditary PGL/PCC syndromes, providing simultaneous analysis of multiple genes that allows faster and more cost-effective mutation detection than previously used Sanger sequencing methods ( 25 , 30 ).

Families 1 and 4 included individuals with head and neck PGLs and individuals with PCCs, with metastatic PCC occurring in both families. The mutated genes differed between the two families with *SDHB* and *SDHD* being affected, respectively. *SDHB* mutations have been associated with extra-adrenal tumors predominantly in the abdomen and pelvis, but with the potential to occur at any location including the adrenal glands, head, and neck. They carry the highest risk of malignancy of all genes associated with hereditary PGL/PCC syndromes. A meta-analysis showed that the pooled prevalence of malignant PGLs in *SDHB* -mutation carriers was 23%, versus only 3% in *SDHD* -mutation carriers; *SDHB* mutations may also predict a shorter survival in persons with malignant PGL/PCC ( 13 , 29 ). The prevalence of germline mutations among children with PGL/PCC tumors in families 1 and 4 underscores the importance of mutation screening in all such families.

The *SDHB* -mutated proband in family 2 was diagnosed with a cervical PGL and had no family history. The mutation site was not a particularly frequent site for *SDHB* mutations, but consistent with Timmers and cols.’s suggestion that *SDHB* -related PGL often presents in sporadic cases ( 32 ). The proband of family 3 had a *SDHD* mutation and all affected individuals of this family had multiple head and neck PGLs, consistent with Benn and cols.’s finding that individuals with *SDHD* mutations have an odds ratio of approximately 24 of developing a skull base or neck PGL compared with individuals with *SDHB* mutations ( 33 ).

The finding of a PCC in a patient with a mutated *SDHD* (family 4 ) can be explained by an established genotype-phenotype association wherein there is a tendency for *SDHD* -nonsense mutation carriers to develop PCC, particularly when the mutation is in the 5’ portion of the gene, as in family 4 ( 34 , 35 ). Interestingly, in family 4, the distribution of individuals with tumors was compatible with maternal transmission, differing from prior reports of tumor development occurring predominantly through paternally inherited *SDHD* mutations ( 16 ). However, this information remains to be corroborated by clinical reports for maternal relatives of the proband in family 4, and genetic testing should be performed to ensure that they are not phenocopies.

Immunohistochemistry (IHC) of SDH subunits can help to identify tumors in *SDH* mutation carriers. When any component of mitochondrial complex II is completely inactivated, the entire complex becomes unstable, resulting in degradation of the SDHB subunit. Therefore, IHC for SDHB is negative whenever SDHA, SDHB, SDHC, SDHD, or SDHAF2 is completely inactivated due to a germline mutation in any of the encoding genes ( 2 ). IHC for SDHA has been also used to reveal *SDHA* mutations ( 36 ). IHC for SDHB has also been used in both familial and (apparently) sporadic PGL/PCC to help guide molecular genetic testing; it has been reported to reduce testing effort, time, and costs ( 30 , 37 , 38 ). However, this strategy was not available for our cases.

Surgical resection is the mainstay treatment for PGL/PCC following appropriate perioperative blockade established in published guidelines ( 23 , 30 , 39 ). However, the surgical approach should be personalized to underlying mutations. Most importantly, PGL/PCC should be resected with great urgency to minimize recurrence and metastasis risk in patients with *SDHB* mutations ( 2 ).

Individuals with germline mutations associated with PGL/PCC should undergo lifelong clinical, biochemical, and imaging surveillance ( 30 , 40 ). Follow-up should be offered to unaffected and affected mutation carriers and relatives at risk based on family history who have not yet undergone genetic testing ( 2 ). Although there are established guidelines to guide surveillance for syndromic (VHL, NF1, and MEN2) PGL/PCC cases, there is not yet a clear consensus for screening carriers of mutations in lower-frequency susceptibility genes, which leaves variance in screening practices based on expert opinion ( 30 , 41 ).

For patients with mutations affecting the SDH complex, surveillance should begin between 5 and 10 years of age; it has been estimated that if screening started at 10 years of age, disease would be detected in all persons with *SDHD* mutations and 96% of persons with *SDHB* mutations ( 33 ). Surveillance should include three facets. Firstly, annual careful history and physical examination, including blood pressure monitoring, should be performed. Secondly, at-risk individuals should be subjected to annual biochemical screening of catecholamine metabolites, known as free metanephrines, in plasma and/or fractionated metanephrines in 24-hour urine samples. Plasma free-methoxytyramine and serum and/or 24-hour urine fractionated catecholamine (dopamine) levels should be evaluated for early detection of cervical PGL. Thirdly, regular imaging studies should be performed; MRI may be the preferable imaging modality to limit radiation exposure ( 11 , 41 ). Some authors have recommended MRI of the abdomen, thorax, and pelvis every 2 years for patients with *SDHB* mutations and MRI of the skull base and neck every 2 years for patients with *SDHC* or *SDHD* mutations; others have recommended MRI from the skull base to the pelvis (or full body if available) every 2 years for all the patients with mutations affecting the SDH complex, as well as for patients with mutations in *TMEM127* or *MAX* ( 2 , 11 , 33 , 39 ). The frequency of imaging studies should be increased in the presence of elevated metanephrine levels and may include functional studies, such as ^123^I-metaiodobenzylguanidine scintigraphy. Regarding other tumors, evaluation for GISTs should be performed in patients with gastrointestinal symptoms, obstruction, or anemia and renal cell carcinoma screening should be performed in patients with *SDHB* mutations ( 2 ).

It has been proposed that genetic testing be offered from the age of 18 years to every person whose parent has a germline *SDHD* mutation, and further suggested that a primary medical evaluation, including imaging, be provided for confirmed *SDHD* carriers ( 40 ). This recommendation includes individuals for whom the affected parent is their mother, despite the aforementioned paternal parent-of-origin effect of *SDHD* mutations because, although rare, there have been cases of PGL/PCC in individuals carrying a germline *SDHD* mutation on the maternal allele ( 40 ).

High-altitude and chronic hypoxia exposure may increase penetrance of hereditary PGL/PCC syndromes ( 34 ). Therefore, lifestyles that promote long-term exposure to hypoxia (e.g. living at a high altitude) and that predispose one to chronic lung diseases (e.g. smoking) should be avoided in individuals with SDH genes or *MAX* mutations ( 2 ). This recommendation was reinforced by recent data showing a gain-of-function somatic mutation of *EPAS1 –* which encodes a transcription factor involved in the physiological response to oxygen concentration changes – in PGL/PCCs of four of five evaluated patients (80%) diagnosed with chronic hypoxemia due to cyanotic congenital cardiopathy ( 42 ).

It is our view that all patients with germline mutations associated with PGL/PCC should undergo genetic counseling regarding familial risk, and that predictive testing of asymptomatic family members be performed when a familial mutation has been identified. Early tumor detection facilitates surgical excision, reduces perioperative morbidity, and can prevent malignant transformation and metastasis ( 2 ).

In conclusion, PGL/PCC tumors are commonly associated with a germline mutation. It is critical that all medical personnel interacting with these patients can recognize signs of inherited cancer predisposition syndromes, because the presence of germline mutations has important implications for treatment, screening, and the surveillance of patients and their family members. These considerations should be incorporated into routine care protocols for patients who have presented with a PGL or PCC.

Individual contributions: A.M.G. and D.C.S. made substantial contributions to the conception and design of this work as well as to data acquisition. A.M.G., A.A.B.C., M.N.F., and M.I.A. participated in the analysis and interpretation of data and the drafting of the manuscript. All authors gave final approval of the submitted version of the manuscript. This manuscript describes original work and is not under consideration by any other journal.
